# Editorial: Imaging of neurometabolism

**DOI:** 10.3389/fnins.2023.1286361

**Published:** 2023-09-15

**Authors:** Wenbin Zheng, Zhouzhi Dai, Renhua Wu, Hongfu Sun

**Affiliations:** ^1^Department of Radiology, The Second Affiliated Hospital, Shantou University Medical College, Shantou, Guangdong, China; ^2^Department of Radiology, Shantou Central Hospital, Shantou, Guangdong, China; ^3^Department of Radiology, Sun Yat-sen Memorial Hospital, Sun Yat-sen University, Guangzhou, Guangdong, China; ^4^School of Information Technology and Electrical Engineering, The University of Queensland, Brisbane, QLD, Australia

**Keywords:** neurometabolism, functional magnetic resonance imaging, magnetic resonance spectroscopy, chemical exchange saturation transfer, diffusion tensor imaging, quantitative susceptibility mapping

Neurometabolic imaging is the study and measurement of neurometabolic activity in the brain using various imaging methods. It is an important field of neuroimaging that has been widely employed in clinical research. Emission computed tomography and fMRI (functional magnetic resonance imaging) are among the methods used. Because of the benefits of non-invasive, non-radiation, and reproducible scanning, fMRI remains the primary imaging tool for the investigation of neurometabolism. This Research Topic contains a collection of papers on the application of neurometabolism-related fMRI methods to clinical disorders, intending to summarize research accomplishments in the field of neurometabolic imaging and probable future paths of development.

Magnetic resonance spectroscopy is a well-established method for quantifying metabolite concentrations in the brain. Wu et al. used ^1^H-MRS (proton magnetic resonance spectroscopy) technology in conjunction with the Mescher-Garwood point resolved spectroscopy sequence to detect metabolite levels in the brain of MRI-negative temporal lobe epilepsy (TLE) patients and discovered γ-Aminobutyric acid to be a potential biomarker for lateralization and monitoring the frequency of epileptic seizures in TLE patients in this Research Topic. However, MRS has long had drawbacks such as low spatial resolution and signal-to-noise ratio, as well as limited resolution of overlapping peaks, whereas chemical exchange saturation transfer imaging, a newer noninvasive method for characterization and quantification of intracerebral metabolites, has high sensitivity and spatial resolution. Zheng H. et al. discovered that the GluCEST (Glutamate Chemical Exchange Saturation Transfer) technique was able to identify brain biochemical changes after acute carbon monoxide poisoning earlier than conventional MRI, and that the CEST technique might be able to compensate for the shortcomings of MRS to some extent. Lin et al. used ^1^H-MRS combined with the GluCEST technique to evaluate the condition and prognosis of patients with acute bilirubin encephalopathy, and discovered that the combination of the two could monitor metabolite levels in the patients' brains and assess the severity of the disease. In a multimodal magnetic resonance study of patients with bipolar disorder and depression, Kong et al. combined ^1^H-MRS, GluCEST, and diffusion kurtosis imaging techniques and discovered that the combination of multiple techniques helped to differentiate between these two disorders. Multimodal magnetic resonance methods will remain appealing and beneficial in the future as one of the avenues of advancement in neurometabolic imaging research.

On the other hand, the invention and refining of novel neurometabolic imaging methods are equally important research avenues. By investigating whether CEST-based k_ex_ (proton exchange rate) MRI (Shaghaghi et al., 2019) combined with quantitative susceptibility mapping could help to further stratify gadolinium-negative multiple sclerosis lesions, Liao et al. validated the role of k_ex_ as a potential biomarker of oxidative stress-induced inflammation. Morita et al., on the other hand, discovered that adolescents' exposure to exercise had potentially negative effects on lymphoid system function in the elderly and was correlated with cognitive decline using the diffusion tensor image analysis along the perivascular space (DTI-ALPS) technique. DTI-ALPS is a new technique capable of quantitatively measuring the function of the lymphoid system proposed by Taoka et al. (2017), and while the technique still has flaws such as low parameter interpretability and overly subjective region of interest selection, some researchers are working on developing methods to address these issues. Dai et al. (2023), for example, developed an FSL-based computer processing algorithm to calculate the ALPS index in order to eliminate subjective measurement error.

In conclusion, this Research Topic article enumerates the use of various types of neurometabolic imaging technologies in clinical research, emphasizes that the cross-use of different technologies and the advancement of new technology development can help to broaden the research space and improve the credibility of experiments, and enumerates the prospects for the use of corresponding technologies in various types of diseases. It serves as a resource for understanding the advancement of neurometabolic imaging techniques and directing future research.

## Author contributions

WZ: Data curation, Project administration, Writing—original draft, Writing—review and editing. ZD: Project administration, Writing—review and editing. RW: Conceptualization, Project administration, Supervision, Writing—review and editing. HS: Project administration, Writing—review and editing.
